# Supplementary material for: Identification of miRNAs Involving Potato-Phytophthora infestans Interaction
Source: Plants (Basel). 2023 Jan 19;12(3):461. doi: 10.3390/plants12030461 (PMC9921761; doi:10.3390/plants12030461)
Supplement: Supplementary file 1 [file plants-12-00461-s001.zip › Figure S1 Vector map.pdf]

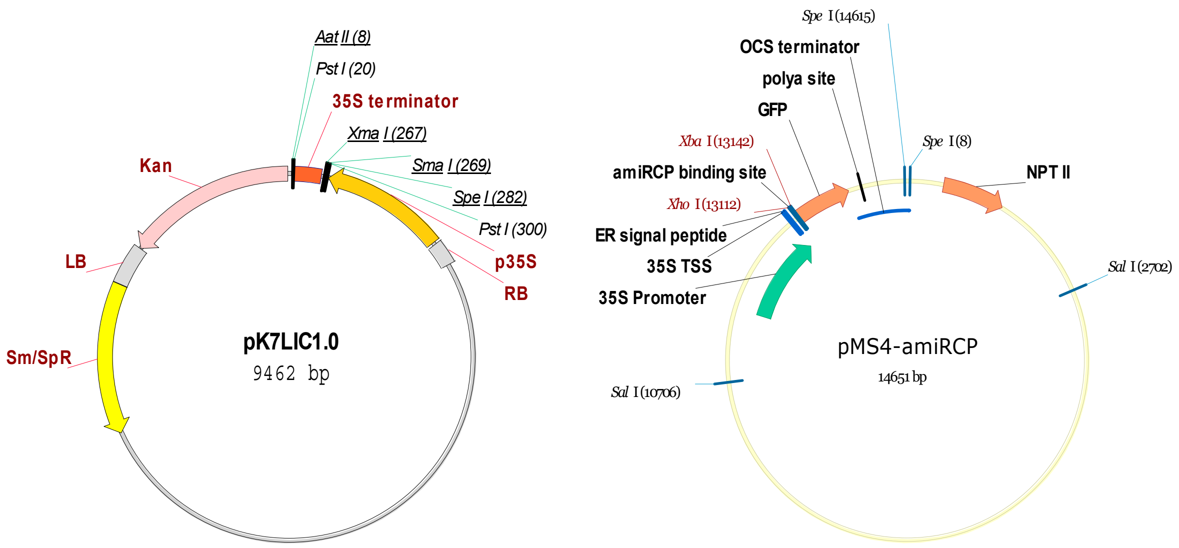

Figure S1. Vector maps used in this study.

Vector pK7LIC1.0 was derived from gateway vector pK7WGF2 (VIB- UGent Center for Plant Systems Biology) replacing its LR recombination Unit and eGFP with a Ligation independent cloning sequences (LIC).

pMS4-amiRCP. The artificial miRNA (amiRCP) binding site is flanked by *Xho* I and *Xba* I restriction sites.
